# Preparation of Skeleton Elements: A Comparative Study on Bone Retrieval by Traditional Burial Method and Modern Chemical Method

**DOI:** 10.7759/cureus.116817

**Published:** 2026-09-24

**Authors:** Shivani Choudhary, Rochita Sharma, Kanika Dhiman, Vandana Tiwari, Richa Niranjan

**Affiliations:** 1 Anatomy, Shri Guru Ram Rai Institute of Medical and Health Sciences, Shri Guru Ram Rai University, Dehradun, IND; 2 Anatomy, Adesh Medical College and Hospital, Shahbad, IND; 3 Anatomy, Shri Mata Vaishno Devi Institute of Medical Excellence, Katra, IND; 4 Anatomy, Government Medical College, Haldwani, IND

**Keywords:** anatomy, bone retrieval, burial method, chemical bleaching method, osteology

## Abstract

Introduction

The foundation of radiology and clinical skills in orthopedics is laid during the teaching of osteology in the anatomy curriculum in medical schools, for which an appropriate bone bank is needed. The retrieval of bones from the discarded cadaveric parts from the previous year's sessions serves as an excellent method of adding new bones to the collection. The present study was conducted to compare the effectiveness of the chemical bleaching method with the burial method in an institute located in a high-humidity environment.

Materials and methods

Two methods were used to retrieve bones: the chemical bleaching method and the traditional burial method. For the chemical bleaching method, the scraped specimens were immersed in a solution of tap water and household bleaching agent (calcium hypochlorite) in the ratio of 5:1 for 24 hours, after which the bones were removed from the solution and scraped again if any tissue was attached to the bone. The bones were then kept in a solution of 6% hydrogen peroxide for 24 hours for whitening, after which the bones were air-dried. For the burial method, one unembalmed body and dissected parts from five embalmed cadaveric specimens were buried in the burial ground for three years. After two years, the burial ground was inspected and excavated; the unembalmed body was totally decomposed, while the embalmed body remained intact with soft tissue requiring a further reburying for another year.

Results

A total of 60 bones were attempted to be retrieved using the chemical method, whereas 800 bones were attempted to be retrieved using the burial method. Thirty-six and 511 bones were successfully retrieved using the chemical and burial methods, respectively. The quality of the bones retrieved is presented as descriptive data.

Conclusions

The chemical bleaching method (calcium hypochlorite) was time-saving and easy to perform for the retrieval of long bones and many short bones. On the other hand, the burial method is useful for the large-scale procurement of bones.

## Introduction

Bones are an integral component of teaching anatomy to medical undergraduate students, and the utility of real bones in understanding the origin and insertion of the muscles and in demonstrating the location of various foramina for different blood vessels cannot be replicated by the artificial bones made using plaster of Paris [1]. There has been a recent mushrooming of new medical colleges in India, and their number has shot up to 750 as of 2025. With the establishment of several new medical colleges across India, there is an urgent need to make bones available for osteology teaching [2].

However, there are several challenges in this context. Firstly, the cost of a single set of real bones could be high, causing an increased financial burden on an institute [1]. Secondly, the sudden demand for bones could create ethical issues with respect to the manner in which bones are retrieved from deceased individuals, including the need to assess the consent and/or coercion of their relatives by exploiting their financial status. This puts the marginalized and poor at a greater risk for exploitation [3].

To address this, several authors have proposed the utilization of technology such as 3D printing for preparing realistic models of bones using materials that closely resemble real bones. These technologies are being explored for application in patient treatment strategies [4], as well as surgical simulations such as middle ear surgeries [5]. However, there is a lack of sufficient data with respect to their utility in teaching osteology to medical students who need access to all bones of the body and not just a particular type of bone such as the temporal bone.

So, the availability of dissected cadavers in medical colleges serves as an excellent cost-effective alternative to retrieve bones for academic teaching. There are several methods of retrieving (also known as bone procurement) bones from the cadavers. The age-old method, that is, the burial method, comprises burying the cadaveric parts in the soil, followed by the retrieval of bones after a period of time. The successful retrieval of quality bones depends upon a lot of factors such as the depth of burial, temperature, and humidity [6]. Other methods include the maceration technique [7], detergent method [8], and boiling method (followed by burial) [8]. Newer methods of chemical action have been explored in the literature recently, and studies have compared the effectiveness of these methods with the classical methods described above [8].

Considering the potential variations in the retrieval of bones by the burial method, the present study was conducted to compare the effectiveness of the chemical bleaching method with the burial method in an institute located in a high-humidity environment.

## Materials and methods

The present study was conducted in the Department of Anatomy at Veer Chandra Singh Garhwali Government Institute of Medical Science and Research, Srinagar, India, between January 2023 and November 2025 using previously dissected, formalin-fixed, human cadaveric parts. The institute being a government-owned and government-run state medical college, the procurement of cadavers is done as per the official protocol of voluntary body donation. Briefly, the required body donation/consent documentation was completed and signed by the donor and/or next of kin, as applicable, and processed through the institutional administration before the acceptance of the donated body for teaching purposes. The cadavers were embalmed according to the standard embalming procedure routinely followed by the Department of Anatomy [9]. The study was conducted after obtaining ethical clearance from the Institutional Ethics Committee (IEC) of Veer Chandra Singh Garhwali Government Institute of Medical Science and Research (vide number MC/IEC/2022-23/56).

The primary outcome was the successful retrieval of skeletal elements using the two methods (chemical and burial), whereas the secondary outcomes comprised the comparison of the two processes in terms of yield rate, duration, and macro-level quality of the retrieved bones. The study included all cadaveric specimens available for bone retrieval by the respective methods during the study period. The number of specimens included in each method was determined by the availability of suitable cadaveric material and the practical feasibility of processing the specimens during the study period. Two different methods for bone retrieval, namely, burial method and chemical bleaching, were used and compared.

Method I (chemical bleaching)

In this method, a total of 60 bones were attempted to be retrieved from the dissected cadaveric parts from the previous five years' dissection sessions that were of no further utility for the current academic session. These specimens were exposed to the chemical action of bleaching powder and hydrogen peroxide (H_2_O_2_) as per the following protocol previously described by Paranjape et al. [10]. The entire procedure was conducted at ambient temperature.

Scraping of Soft Tissue

The loose muscle and ligaments were gently scraped from the dissected cadaveric parts using a scalpel. Scraping was done from the following specimens: foot (n=1), thigh (n=5), lower leg (n=4), hip (n=3), skull vault (n=6), scapula (n=3), clavicle (n=2), and upper limb (n=1).

Bleaching Agent

The purpose of the bleaching agent was to degrease the bones and soften the tissue still adherent to the bones after the above step of removing the soft tissue using a scalpel. A solution of tap water and bleaching agent, that is, calcium hypochlorite (GRM1223, HiMedia Laboratories, India), was prepared in a ratio of 5:1, and bones were immersed in this solution for up to 24 hours, after which a second session of scraping was done using a scalpel. The brushing of the bones was further undertaken to impart them a clean, tissue-free look. The bones were finally washed with detergent before initiating the next step.

Degreasing

The aim of this process was the removal of oil/grease from the bone. In the present study, acetone was used as a degreasing agent, and all bones (n=36) were dipped in approximately 15 L of acetone solution for 12 hours, after which they were kept in air for drying. Degreasing is done to remove the fat secreted by the bone marrow of the bone. If the process of degreasing is not done, the bone attracts more dirt and appears oily. The use of acetone warranted careful fire and chemical hazard risks. Acetone was added to a metal container with a lid and placed in a separate room away from the critical infrastructure of the department and was sealed tightly to avoid fumes that could increase the risk of fire in the room. Personnel used eye protection/goggles, a face mask, and rubber gloves to avoid contact with acetone. The chemical left after the step was handed over to the designated authorized personnel responsible for handling chemical and biomedical waste in the facility.

Hydrogen Peroxide

The bones were immersed in 6% hydrogen peroxide solution for 24 hours for bleaching (which helps in bone whitening) to give them a neat/clean look.

Drying

The bones were then air-dried (slow drying) for three weeks, depending upon the weather conditions. The air-drying helped remove the moisture from the bones, which is detrimental to their durability and could make them soft over time.

Finishing

In the final step, the bones were painted using turpentine oil to improve their durability and reduce the incidence of bone infestation with insects/pests.

Method II (burial method)

Around five to six years old, disarticulated parts of five cadavers were taken for the burial method; along with these parts, one unembalmed body was also buried in the burial ground. All such formalin-fixed cadaveric parts and one unembalmed body were buried in the burial ground for a period of approximately three years.

Burial Using Loam and Salt

The burial ground was filled with loamy sand that allows the atmospheric air to pass through the various strata of soil and facilitate the decomposition process. All these cadaveric parts and one unembalmed body were buried at a 2 ft depth in the burial ground. Around 8 kg of common raw salt was also spread over the dissected cadaveric parts at the time of burial in order to reduce the moisture and minimize unpleasant odors associated with decomposition. The site of burial was inspected every six months to ensure its integrity.

Extraction of the Bone

The burial ground was inspected and excavated after two years. The bones of the unembalmed body were free from soft tissue within two years; these bones were retrieved effectively, but the formalin-fixed parts still had soft tissue attached to the bones; these parts were reburied in the burial ground. After the completion of one more year, the burial ground was excavated; this time, the soft tissue attached to the bone was completely decomposed, and the bones were retrieved efficiently. So, the retrieval of the bone from a formalin-fixed specimen took three years to decompose.

Bone Whitening

After the retrieval of the bone from the burial ground, the bones were treated with 6% hydrogen peroxide.

Drying

The bones were air-dried for three weeks in the winter season.

Finishing

Finishing was done using turpentine oil and by drying them.

Quality assessment of retrieved bones

The quality of the retrieved bones was assessed descriptively with respect to their suitability for the academic teaching of osteology. The assessment considered the general appearance, preservation of the anatomical features, and overall suitability of the specimens for teaching purposes. In addition, the retrieved bones were shown to phase year-1 (PY-1) MBBS students nearing the completion of their academic year, and their feedback regarding the perceived academic utility and suitability of the specimens was obtained. No validated or standardized instrument was available or developed for this assessment (refer to "Limitations" under the Discussion section).

## Results

A total of 60 bones were attempted to be retrieved using the chemical method, whereas 800 bones were attempted to be retrieved using the burial method. The chemical method yielded 36 bones, including long bones, irregular bones, and small bones. Foot bones were the most frequently retrieved skeletal elements by this method (n=14), followed by the femur (n=5), tibia (n=4), hip (n=3), and fibula (n=2). In the burial method, 470 bones were retrieved from embalmed cadaveric material and 41 from the unembalmed cadaver. Among the embalmed material, foot bones (n=100), hand bones (n=95), vertebrae (n=95), and ribs (n=98) accounted for the largest numbers, whereas long bones such as the radius, femur, tibia, and humerus were also retrieved in appreciable numbers. From the unembalmed cadaver, vertebrae (n=13) and ribs (n=12) were the most frequently recovered elements. The distribution of the individual skeletal elements retrieved by each method is presented in Table 1.

**Table 1:** Bones retrieved by the chemical and burial methods

Bone	Chemical (n {%})	Burial	
		Embalmed (n {%})	Unembalmed (n {%})
Humerus	1 (100)	9 (90)	2 (100)
Radius	1 (100)	10 (100)	1 (50)
Ulna	0 (0)	8 (80)	1 (50)
Femur	5 (100)	10 (100)	2 (100)
Tibia	4 (100)	10 (100)	2 (100)
Fibula	2 (100)	8 (80)	2 (100)
Hip	3 (100)	8 (80)	2 (100)
Sacrum	1 (100)	4 (80)	1 (100)
Skull (excluding vault)	0 (0)	3 (50)	0 (0)
Skull vault	3 (50)	6 (100)	1 (100)
Vertebrae	0 (0)	95 (79.2)	13 (54.2)
Ribs	0 (0)	98 (84.9)	12 (50)
Clavicle	1 (50)	3 (37.5)	0 (0)
Scapula	1 (33.3)	3 (37.5)	2 (100)
Hand bones	0 (0)	95 (70.4)	0 (0)
Foot bones	14 (53.8)	100 (76.9)	0 (0)
Total	36 (60)	470 (79.1)	41 (23.4)

Descriptive data of the retrieval process

Long, dense bones such as the femur, humerus, radius, tibia, and fibula were the easiest to retrieve and required the least amount of effort and time during the retrieval process by the chemical method (Figure 1). Irregular bones and small bones such as the pelvis and tarsal and metatarsal bones of the foot needed extra effort and time for their retrieval but were effectively retrieved. However, bones such as the scapula, clavicle, and ribs posed significant challenges in their retrieval by the chemical method, as roughly half of these bones (the scapula {n=2}, ribs {n=8}, and clavicle {n=}) lost their strength and rigidity, becoming soft and somewhat friable, rendering them unsuitable for osteology teaching. The challenges in retrieving vertebrae were mainly due to the difficulty in clearing soft tissue around the vertebral foramina and transverse foramina.

**Figure 1:**
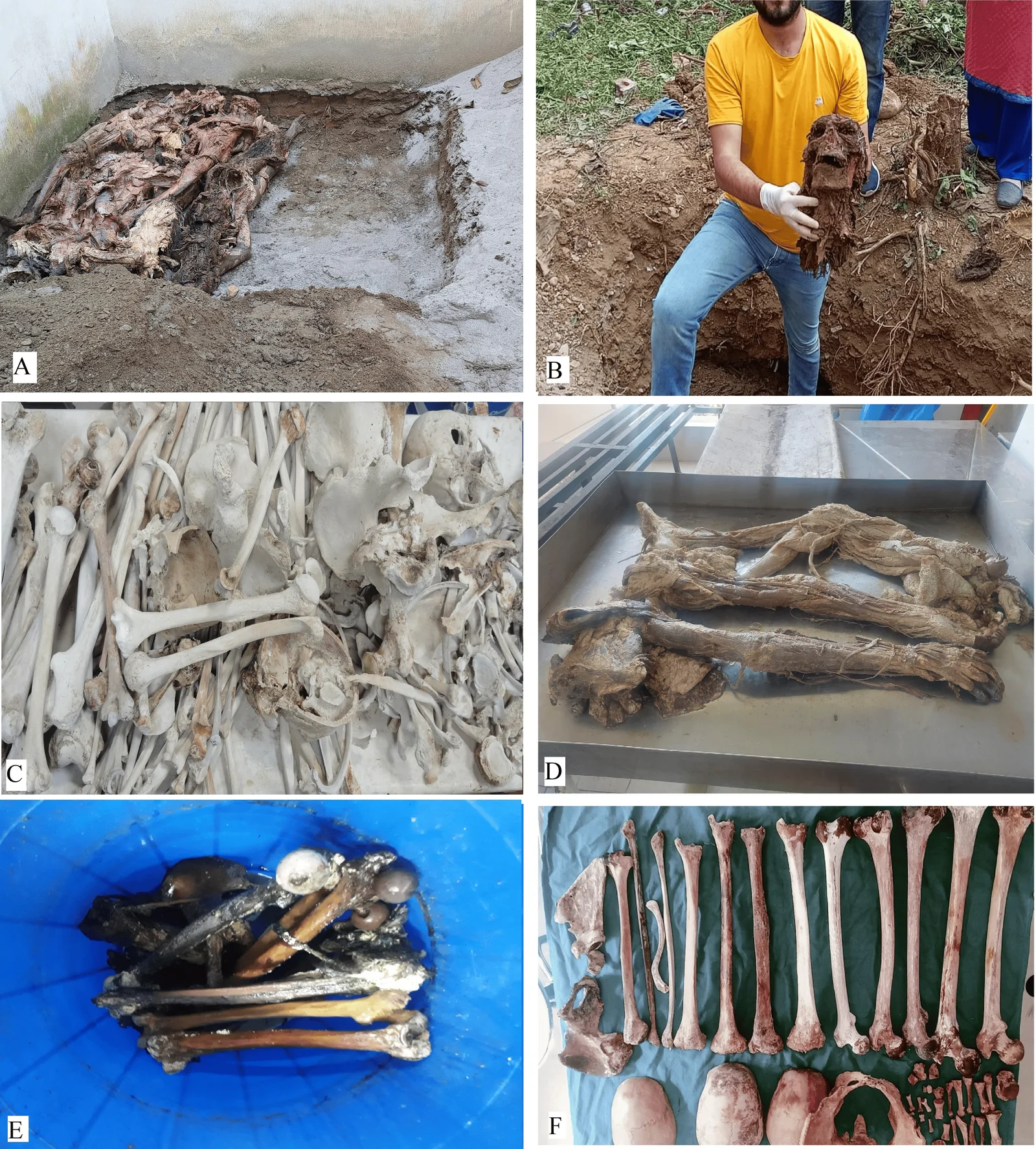
Bones retrieved using the burial method (A, B, and C) and chemical method (D, E, and F) A, deep burial of discarded cadaveric parts and an unembalmed body; B, retrieval of an unembalmed body part after two years of deep burying; C, retrieved bones from the burial method; D, discarded cadaveric parts before the chemical retrieval process; E, retrieved bones by chemical method before the hydrogen peroxide step (refer to step "Hydrogen Peroxide" in Method I under the Materials & Methods section); and F, retrieved bones using the chemical method after hydrogen peroxide cleaning

Five cadaveric disarticulated parts, along with one unembalmed body, were buried in the burial ground at a 2 ft depth for three years. The soil used for the burial ground was loamy in nature. Around 8 kg of common raw salt was sprinkled during the burial of these parts; common salt is hygroscopic in nature, resulting in wet-dry strata of soil. After the completion of two years, the burial ground site was inspected and excavated; the soft tissue around the bones of the unembalmed cadaver was effectively removed, and the bones were freed from soft tissue within two years. A total of 41 bones were retrieved from one unembalmed cadaver. The skull bone of the unembalmed cadaver was not intact; it was found in four parts, as the skull bone was eaten by maggots present in the soil.

On the contrary, bones from formalin-fixed cadaveric parts still had remaining soft tissue adherent to their surface. Thus, they were again reburied in the loamy soil, and additionally, a bag of sand weighing approximately 50 kg was added to the soil to make the soil maggot-free, and the burial period was extended for one additional year. A total of 800 bones were expected to be recovered from the burial ground, but 289 bones were lost, including the ribs, scapula, cervical vertebrae, hand bones, foot bones, and some parts of the skull. The epiphyseal ends were decomposed in roughly 30 bones, which were otherwise retrieved intact in the context of the rest of their parts. The observations are summarized in Table 2.

**Table 2:** Comparison of bleaching with burial method ^#^The color of the retrieved bone after the completion of the processing *Quantity of bones that can be processed per batch

Parameter	Bleaching method	Burial method (embalmed)	Burial method (unembalmed)
Duration	1 month and five days	Three years	Two years
Color^#^	Brownish	Yellowish white	Yellowish white
Damaging effects	No damage in long bones but some bones such as the scapula and ribs were damaged	A large number of bones were intact with no damage, but the epiphyseal end of some bones got reabsorbed	A large number of bones were intact with no damage, but skull bones were found disarticulated
Odor	No odor	No odor	No odor
Quantity*	Small	Large	Large

## Discussion

The suitability and feasibility of adopting a bone retrieval technique in an institute are determined by the time taken, the resources required, and the percentage of bones successfully retrieved in relation to the intended purpose for which the retrieval is done. However, the boiling technique is discouraged in modern practice as it results in the loss of the structural integrity of the bone [11], besides producing hazardous fumes and causing potential environmental damage [7]. The average time required for the retrieval of bones by the burial method is approximately two years and is dependent upon the environmental factors and soil bacteria [6]. Standard reported methods for retrieving the bone are either boiling or burial. This time period is even longer and could even be unsuccessful when cadaveric parts from embalmed cadavers were buried for bone retrieval. It is agreed that the decomposition of soft tissue is delayed in embalmed bodies due to the presence of formalin, but boiling before the burial of such specimens leads to the enhancement of soft tissue decomposition due to a decrease in formalin after boiling [12].

In the present study, bone retrieval was done using two methods, namely, the chemical bleaching method and the burial method, using formalin-fixed, discarded cadaveric parts. The burial technique was selected because of its eco-friendly nature, cost-effectiveness and minimal resources, and no usage of harsh chemicals that could pose a hazard to both the user and the environment [13]. Another important advantage of this method is its ability to preserve the natural color and integrity of the bones, causing no shrinkage or damage, as compared with other methods such as boiling or chemical maceration [14].

The disadvantage of the burial method, as noted in the present study, was its time-consuming nature since it required months to years for the complete decomposition of the soft tissue. For this process, a burial ground is required, as it is a land-dependent process. Finding a suitable site posed challenges in the present study due to space constraints for an institute located in an urban town. Other factors influencing the quality of bone retrieval include the composition of soil, depth of the burial ground, and environmental conditions such as environmental humidity and temperature [15].

The use of chemicals for the retrieval of bones should be undertaken carefully [16]. Detrimental effects of using sodium hypochlorite for bone cleaning and degreasing have been described, which cause collagen breakdown and the subsequent loss of the integrity of the bone strength (eventually leading to bones turning into powder). The bones retrieved by the chemical method in the first lot in February 2023 in the present study showed no such loss of integrity as of December 2025. These bones have been utilized by the undergraduate students from the last three batches, indicating that the chemical method is an effective, practical, and rapid technique that makes the bone free from soft tissue without producing any unpleasant odor and can be done in the Department of Anatomy.

However, the chemical method used in the present study failed to retrieve the facial skull bone, vertebrae, scapula, and ribs. It was observed that the foramina in the skull bone and vertebrae were difficult to clean since soft tissue was tightly attached in these locations. The possible reason for the difficult retrieval of the scapula and ribs could be attributed to the fact that these specimens were kept preserved in formalin for a long period, thereby making them brittle and leading to changes in bone density. A similar observation was reported by Paranjape et al., who recommended the use of fresh cadaveric specimens for retrieving the bones since they were unable to retrieve the scapula and hand bones as the specimens were three to four years old. Another disadvantage of the chemical bleaching method noted in the present study was the use of acetone for the degreasing of the bone [1]. Acetone is a highly volatile organic compound that is miscible with water and is flammable. It should be used with caution, and its storage and disposal warrant an equally cautious approach due to its combustible nature. A comparison of the methodologies and the key observations reported in the previous literature is presented in Table 3.

**Table 3:** Methodology and key findings reported from previous studies H_2_O_2_: hydrogen peroxide

Author	Method	Key observations
Modi et al. (2014) [12]	Used four different methods: (a) boiling method, (b) dipping bone in warm water, (c) partially cleaned bone in warm water, and (d), after boiling, the bone was buried in the burial ground	Burial, followed by boiling, was the most effective method of bone retrieval
Aggarwal et al. (2016) [17]	Used the burial method in combination with different concentrations of hydrogen peroxide as given in the following: (a) ratio of 1:0.5 (H_2_O_2_ and water), (b) ratio of 1:1 (H_2_O_2_ and water), (c) ratio of 1:2 (H_2_O_2_ and water), and (d) ratio of 1:3 (H_2_O_2_ and water)	Burial, followed by treatment with 1:2 hydrogen peroxide and water, produced the best results for bone procurement. The procedure rendered the bones devoid of soft tissue in approximately 18 hours with this method
Mishra et al. (2016) [6]	Comparison of varying depth of burial (1 ft, 2 ft, 3 ft, and 4 ft) on the effectiveness of bone retrieval	Burial at a 2 ft depth was the most effective and practical method for bone retrieval
Paranjape et al. (2017) [10]	Use of washing soda and hydrogen peroxide combination	Retrieved a total of 27 bones in 30-36 working hours with well-preserved morphological features
Odukoya et al. (2017) [14]	Used two different methods: (a) cold-water maceration and (b) hot-water maceration methods	The quality of the bone retrieved by cold-water maceration was best in terms of color and morphological features of the bone; the duration for this process was two months and 15 days, while for hot-water maceration, the duration was 2-3 weeks; the bone became reddish-brown in color, and many bones became shrunken, and some morphological features were also missing
Sarma et al. (2017) [18]	Retrieved bone by three methods. Manual cleaning was followed by one of the following three: (a) Group I is treated with ascending concentrations of chlorine solution (50%, 70%, and 90%), (b) Group II is treated with water, and (c) Group III is buried in the burial ground for 30 days	The use of chlorine solution for cleaning bones gives the best results among the three
Soni et al. (2021) [7]	Compared four different methods: (a) burial, followed by detergent treatment; (b) burial, followed by the use of quick lime solution; (c.) burial, followed by treatment with detergent and 30% H_2_O_2_;and(d) using a paste of 100 g baking soda and 30% H_2_O_2_	The concentration of 30% H_2_O_2_ following burial was the most effective, requiring lesser time for soft tissue maceration and removal and lower adverse effects on the morphological characteristics of the bones
Shahabuddin et al. (2023) [19]	Used four different methods: (a) burial, followed by detergent; (b) burial, followed by dipping the bone in liquid lime solution; (c) burial, followed by use of 30% H_2_O_2_; and (d) using a paste of baking soda along with 30% H_2_O_2_	The concentration of 30% H_2_O_2_ was the best among the four tried methods. The bones were cleaned effectively; there was no damage to the bone. The disadvantage of this method is that it requires more time

Limitations

The study has a few limitations. A limitation of the study was the unequal number of bones subjected to the two retrieval methods. This difference was primarily due to the practical constraints of implementing the chemical method in relatively small batches, whereas a larger quantity of discarded cadaveric material could be collectively subjected to the burial method. Therefore, the findings should be interpreted in the context of the actual operational conditions under which the two methods were evaluated. Another limitation was the absence of a validated or standardized instrument for assessing the academic suitability and perceived quality of the retrieved bones. The present assessment was based on descriptive observations and student feedback; the development and validation of a standardized assessment tool would allow more objective evaluation in future studies.

## Conclusions

The present study provides data on the optimum utility of the two methods, that is, burial and chemical methods, based on user requirements. The burial method is a more suitable method for the retrieval of bones as compared with the chemical method. It is also a cost-effective and safe method but requires more space and time. For quicker retrieval, the chemical method offers a more suitable alternative, with the limitation that the method is mainly suitable for the long bones. The choice should be guided by the intended application of the specimen, as well as available resources and institutional safety guidelines.
